# Supplementary material for: Mechanisms of substrate recognition by a typhoid toxin secretion-associated muramidase
Source: eLife. 2020 Jan 20;9:e53473. doi: 10.7554/eLife.53473 (PMC6996933; doi:10.7554/eLife.53473)
Supplement: Supplementary file 1. — Values in parenthesis are for the highest resolution shell. [file elife-53473-supp1.docx]

**Supplementary File 1.** Data Collection and refinement statistics. Values in parenthesis are for the highest resolution shell.

| **Structure** | **TtsA** | **Sen1395** |
| --- | --- | --- |
| **Data Collection** |  |  |
| Wavelength (Å) | 0.97936 | 0.97920 |
| Space group | C 2 2 21 | P1 |
| Cell dimensions |  |  |
| a, b, c (Å) | 136.9 138.61 135.20 | 35.37, 49.37, 58.64 |
|    º | 90, 90, 90 | 91.41, 107.55, 93.61 |
| Molecules/ASU | 4 | 2 |
| Resolution (Å) | 43.37 - 2.10 (2.18 - 2.10) | 36.12 - 1.90 (1.97 - 1.90) |
| Rmerge | 0.068 (0.964) | 0.064 (0.572) |
| *I* / *I* | 22.4 (1.3) | 11.3 (2.0) |
| Completeness (%) | 98.3 (86.7) | 89.9 (80.3) |
| Redundancy | 4.9 (4.8) | 2.6 (2.3) |
| Unique reflections | 73442 (6414) | 26682 (2484) |
| CC1/2 | 0.891 (0.462) | 0.980 (0.376) |
|  |  |  |
| **Refinement** |  |  |
| # of non-hydrogen atoms | 6501 | 2885 |
| Rwork/Rfree (%) | 19.0/22.6 (29.2/30.5) | 20.0/24.9 (41.6/46.1) |
| Average B factor (Å2) | 52.9 | 63.7 |
| Root mean-squared deviation (rmsd) |  |  |
| Bond lengths | 0.010 | 0.008 |
| Bond angles | 1.3 | 1.2 |
| Ramachandran analysis |  |  |
| Preferred regions (%) | 95.9 | 94.9 |
| Allowed regions (%) | 4.1 | 4.0 |
| Outliers (%) | 0.0 | 1.1 |

1
