# Supplementary material for: Mechanisms of substrate recognition by a typhoid toxin secretion-associated muramidase
Source: eLife. 2020 Jan 20;9:e53473. doi: 10.7554/eLife.53473 (PMC6996933; doi:10.7554/eLife.53473)
Supplement: Supplementary file 2. [file elife-53473-supp2.docx]

| **Strains** | **Description** | **Reference** |
| --- | --- | --- |
| SB 2201 | *S*. Typhi WT ISP2825 reference strain | (*1*) |
| SB 1946 | *S*. Typhi *cdtB^3xFlag^* | (*2*) |
| SB 1949 | *S*. Typhi *∆ttsA cdtB^3xFlag^* | (*3*) |
| SB 1844 | *S*. Typhi *∆ttsA* | (*3*) |
| SB 3618 | *S*. Typhi *ttsA (N166D) cdtB^3xFlag^* | This study |
| SB 3614 | *S*. Typhi *sen1395 cdtB^3xFlag^* | This study |
| SB 3615 | *S*. Typhi *sen1395 (D167N) cdtB^3xFlag^* | This study |
| SB 3617 | *S*. Typhi *sen1395 (1-92) ttsA (93-180) cdtB^3xFlag^* | This study |
| SB 3245 | *S*. Typhi *ttsA^3xFlag^* | (*4*) |
| SB 3613 | *S*. Typhi *ttsA (N166D)^3xFlag^* | This study |
| SB 3616 | *S*. Typhi *sen1395^3xFlag^* | This study |
| SB 3608 | *S*. Typhi *sen1395 (D167N)^3xFlag^* | This study |
| SB 3619 | *S*. Typhi *sen1395 (1-92) ttsA (93-180)^3xFlag^* | This study |
| SB 3301 | *S*. Typhi *ttsA (E14::TAG)^3xFlag^* | This study |
| SB 3300 | *S*. Typhi *ttsA (D118::TAG)^3xFlag^* | This study |
| SB 3247 | *S*. Typhi *ttsA (N166::TAG)^3xFlag^* | This study |
| SB 3602 | *S*. Typhi *ttsA (F169::TAG)^3xFlag^* | This study |
| SB 3308 | *S*. Typhi *∆ycbB cdtB^3xFlag^* | (*4*) |
| BL21 (DE3) | *E. coli* protein expression strain | Invitrogen |
| β-2163 | *E. coli* *∆nic 35* | (*5*) |
| **Plasmids** |  |  |
| pSB 890 | R6K origin, counter selectable with sucrose, mutagenesis plasmid | (*6*) |
| pSB 5964 | pSB890 *ttsA (N166D)* to mutate *ttsA* in the chromosome | This study |
| pSB 5920 | pSB890 *sen1395* to replace *ttsA* in the chromosome | This study |
| pSB 5961 | pSB890 *sen1395 (D167N)* to replace *ttsA* in the chromosome | This study |
| pSB 5971 | pSB890 *sen1395 (1-146) ttsA (147-180)* to replace *ttsA* in the chromosome | This study |
| pSB 5970 | pSB890 *sen1395 (1-92) ttsA (93-180)* to replace *ttsA* in the chromosome | This study |
| pSB 6044 | pSB890 *ttsA (N166D)^3xFlag^* to replace *ttsA* in the chromosome | This study |
| pSB 5974 | pSB890 *sen1395^3xFlag^* to replace *ttsA* in the chromosome | This study |
| pSB 6045 | pSB890 *sen1395 (D167N)^3xFlag^* to replace *ttsA* in the chromosome | This study |
| pSB 6046 | pSB890 *sen1395 (1-92) ttsA (93-180)^3xFlag^* to replace *ttsA* in the chromosome | This study |
| pSB 5430 | pSB890 *ttsA (E14::TAG)^3xFlag^* to mutate *ttsA* in the chromosome | This study |
| pSB 5429 | pSB890 *ttsA (D118::TAG)^3xFla^* to mutate *ttsA* in the chromosome | This study |
| pSB 5466 | pSB890 *ttsA (N166::TAG)^3xFlag^* to mutate *ttsA* in the chromosome | This study |
| pSB 5438 | pSB890 *ttsA (F169::TAG)^3xFlag^* to mutate *ttsA* in the chromosome | This study |
| pSB 3783 | pBAD24 with tetracycline resistance, used for trans complementation and protein expression studies | (*4*) |
| pSB 5904 | pSB3783 *ycbB* | (*4*) |
| pSB 5906 | pSB3783 *ttsA^3xFlag^* | (*4*) |
| pSB 5913 | pSB3783 *sen1395^3xFlag^* | This study |
| pSB 5917 | pSB3783 *ttsA* *(N166D)^3xFlag^* | This study |
| pSB 5915 | pSB3783 *sen1395 (D167N)^3xFlag^* | This study |
| pSB 6042 | pSB3783 *sen1395 (1-92) ttsA (93-180)^3xFlag^* | This study |
| pSB 6043 | pSB3783 *ttsA (E14A)^3xFlag^* | This study |
| pSB 5975 | pSB3783 *ttsA (I116D)^3xFlag^* | This study |
| pSB5978 | pSB3783 *ttsA (D118A)^3xFlag^* | This study |
| pSB 5976 | pSB3783 *ttsA (Q120R)^3xFlag^* | This study |
| pSB 6041 | pSB3783 *ttsA (Q164E)^3xFlag^* | This study |
| pET 28b | protein expression vector | Invitrogen |
| pSB 5485 | pET28b *ttsA*-N term. histidine tag | (*4*) |
| pSB 5967 | pET28b *sen1395*-N term. histidine tag | This study |

Table S2: Strains and plasmids

1. J. E. Galan, R. Curtiss, 3rd, Distribution of the invA, -B, -C, and -D genes of Salmonella typhimurium among other Salmonella serovars: invA mutants of Salmonella typhi are deficient for entry into mammalian cells. *Infection and immunity* **59**, 2901-2908 (1991).

2. S. Spano, J. E. Ugalde, J. E. Galan, Delivery of a Salmonella Typhi exotoxin from a host intracellular compartment. *Cell Host Microbe* **3**, 30-38 (2008).

3. H. Hodak, J. E. Galan, A Salmonella Typhi homologue of bacteriophage muramidases controls typhoid toxin secretion. *EMBO Rep* **14**, 95-102 (2013).

4. T. Geiger, M. Pazos, M. Lara-Tejero, W. Vollmer, J. E. Galan, Peptidoglycan editing by a specific LD-transpeptidase controls the muramidase-dependent secretion of typhoid toxin. *Nature microbiology* **3**, 1243-1254 (2018).

5. G. Demarre *et al.*, A new family of mobilizable suicide plasmids based on broad host range R388 plasmid (IncW) and RP4 plasmid (IncPalpha) conjugative machineries and their cognate Escherichia coli host strains. *Research in microbiology* **156**, 245-255 (2005).

6. K. Kaniga, J. C. Bossio, J. E. Galan, The Salmonella typhimurium invasion genes invF and invG encode homologues of the AraC and PulD family of proteins. *Molecular microbiology* **13**, 555-568 (1994).
